# A Multi-level Remedial Teaching Design Based on Cognitive Diagnostic Assessment: Taking the Electromagnetic Induction as an Example

**DOI:** 10.3389/fpsyg.2022.851378

**Published:** 2022-03-23

**Authors:** Rui Huang, Zengze Liu, Defu Zi, Qinmei Huang, Sudong Pan

**Affiliations:** ^1^School of Physics and Electronic Science, East China Normal University, Shanghai, China; ^2^College of Teacher Education, Faculty of Education, East China Normal University, Shanghai, China; ^3^Dali No. 1 High School, Dali, China

**Keywords:** multi-level teaching, remedial teaching, electromagnetic induction, DINA model, cognitive diagnostic assessment

## Abstract

Multi-level teaching has been proven to be more effective than a one-size-fits-all learning approach. This study aimed to develop and implement a multi-level remedial teaching scheme in various high school classes containing students of a wide range of learning levels and to determine its effect of their learning. The deterministic inputs noisy and gate model of cognitive diagnosis theory was used to classify students at multiple levels according to their knowledge and desired learning outcomes. A total of 680 senior high school students from central provinces in China participated in the initial cognitive diagnostic test, and 1,615 high school sophomores from seven high schools in China participated in a formal cognitive diagnosis test. Thirty-six high school students from Southwestern China participated in the think-aloud protocols, and 258 seniors from three high schools in southwest China participated in the remedial teaching experiment. Through an analysis of students’ think-aloud protocols, cognitive errors of students at all levels were determined, and multi-level remedial teaching programs were designed to address these common cognitive errors. The remedial teaching programs were then implemented in three schools and compared with a control group. The results indicated that the students in the experimental group showed a more significant improvement. In this study, the steps of designing multi-level remedial teaching include assessment, classification, and preparing a teaching scheme, which are feasible and can have remarkable teaching effects. This process can be used for reference by teachers of various subjects.

## Introduction

Psychometry-based cognitive diagnostic assessment (CDA) is a method that can be used by frontline teachers to determine students’ learning outcomes and classify them based on their diagnostic results for more personalized remedial teaching. At the core of the new generation of test theory are cognitive diagnosis models (CDMs)—models which are able to measure more detailed information about participants’ knowledge, skills, and strategies. Currently, research on cognitive diagnosis has received worldwide attention from researchers, teachers, and evaluators alike, with a number of studies having implemented individualized remedial teaching for students based on diagnostic classifications.

One study by Wang et al. (2021) applied the CDM to evaluate and classify urban and rural middle school students based on their mastery of a topic on linear equations. Remedial teaching based on cognitive results was implemented for the test group, while the traditional “answer-explanation” style of remedial teaching was implemented for the control group. Results showed that remedial teaching based on cognitive diagnosis results significantly improved the learning effect. Similarly, Ren et al. (2021) used the CDM to learn about the poorly mastered attributes of “data distribution characteristics” in teaching math to middle schoolers and found that verified cognitive diagnoses can be used for targeted interventions to improve students’ abilities more effectively. Consistent with this, Fan et al. (2021) proposed an integrative framework of diagnosis which connects CDA to feedback and remediation, and they empirically demonstrated the application of the framework in an English as a Foreign Language (EFL) context.

These studies suggest that CDA can effectively diagnose students’ learning outcomes and be used to conduct personalized remedial teaching. However, the basis of remedial teaching in these studies was only whether or not attributes were mastered, but the level of mastery of attributes was not graded. With this being said, in order to be truly personalized, the basis for classification in remedial teaching should not only be dependent on whether the attribute is mastered, but also on the mastery level of knowledge, skills, and cognitive processes. Hence, in this study, multi-level remedial teaching refers to the remedial teaching for students based on their respective levels of knowledge or skills mastery for a specific topic after they have learned it in class.

Multi-level teaching and learning are widely regarded as an important way of improving teaching efficiency. In recent years, many studies have designed and developed methods to stratify students’ learning outcomes, with many of these methods in previous literature being based on computer algorithms (He et al., 2016; Zhang et al., 2016). For instance, Wu (2019) used CDA to classify fourth grade students based on their learning scores in order to provide personalized online remedial guidance, and results showed that the online personalized tutor program was superior to the traditional tutorial program. You et al. (2019) applied CDA’s deterministic inputs noisy and gate (DINA) model to develop a cognitive diagnostic system for Chinese learning, which was applied in all subjects of an experimental high school to provide personalized learning feedback for students and teachers. The study found that through this the efficiency and self-efficacy of students improved. Additionally, Shute et al. (2008) developed a multivariate probit model for CDA and applied it to the data from the Adaptive Content with Evidence-Based Diagnosis (ACED) evaluation study to verify the validity of the new model. These aforementioned studies developed adaptive learning systems based on cognitive CDA. However, to be applied in schools, this requires the purchase of hardware and software which may be difficult to use for some frontline teachers.

Computer-based adaptive learning is a process dependent on the use of a technological device, whereas teacher-student interaction is much more common in Chinese high schools. Therefore, our study provides frontline teachers with examples of multi-level teaching designs based on CDA without the need for large hardware or paid software services and integrates experiments and discussions into multi-level remedial teaching that human-computer interactions cannot provide.

Most of the abovementioned CDA application cases in educational practice are conducted in subjects such as mathematics (Groß et al., 2016) and second foreign languages (Liu et al., 2013), but its application in the sciences have not yet been thoroughly studied. Zhan et al. (2019b) developed a new CDM which realized the assessment of scientific literacy and filled the gap in the application of CDA in scientific disciplines. Practical data from an eighth-grade physical circuit topic were used to examine the performance of the newly developed model (Zhan et al., 2019a). However, there is currently almost no empirical remedial teaching research on applying CDMs to the physics curriculum in high school education.

The present study aimed to design and implement a micro multi-level remedial teaching plan based on CDA that is easy to use in the classroom-setting and which may be applied to high school physics remedial teaching plans. Electromagnetic induction is a very important chapter in high school physics. It is inherently logical and difficult to learn. This study takes the topic of electromagnetic induction as an example. This study intended to solve the following problems:

1. How can multi-level classifying be performed for students learning a certain topic?
2. How can a multi-level remedial teaching plan be developed according to the created classifications?
3. Does the implementation of multi-level remedial teaching effectively improve students’ learning results?

## Materials and Methods

**Figure 1:**
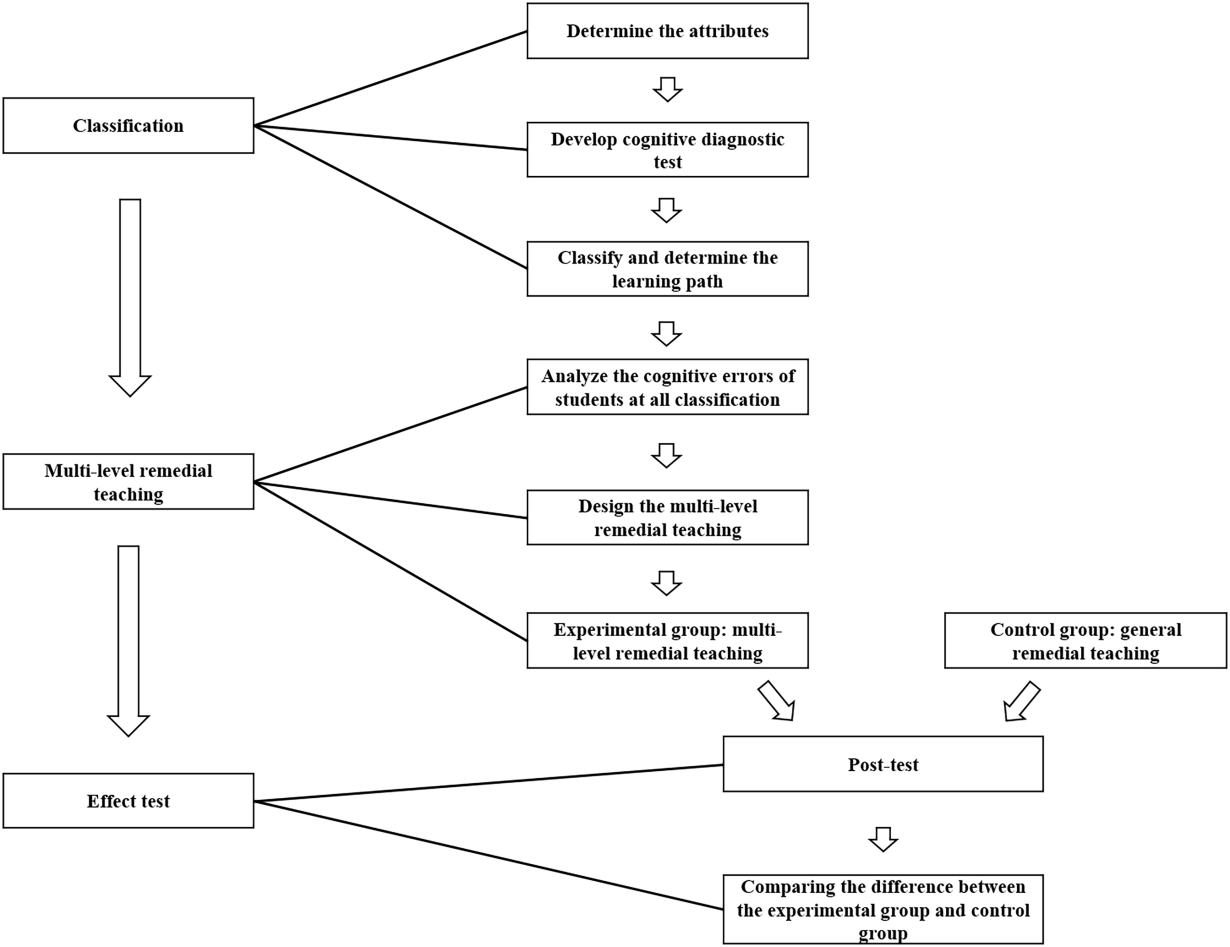
The multi-level remedial teaching research process.

In this study, CDM was used to assess and classify students’ learning of electromagnetic induction topics in high school. To design multi-level remedial teaching, think-aloud protocols of students at different levels were collected to analyze their thinking and determine common cognitive errors of students at each level. Multi-level remedial teaching plans were then designed. After the multi-level remedial teaching experiment, the students completed a post-test, which was used to test the intervention effect. The research process is shown in Figure 1.

### Assessment and Classification

The CDM was used to assess students’ learning on electromagnetic induction. Based on the results of the assessment, the students were classified according to their level of learning of concepts and rules.

#### Multi-level Attributes

For the hierarchical diagnostic classification of learning result, attributes will be identified by two dimensions: knowledge content and learning level.

In this study, to determine the attributes, five physics teachers with over 10 years of teaching experience in high school physics were invited to analyze the content and the learning result levels determined based on the questions in the question bank. The bank contained a total of 53 multiple-choice items on electromagnetic induction from five recent versions of the Chinese Higher Education Entrance Examination (2014–2018) and academic level examination review papers. According to the teachers, the question bank contents related to the topic of electromagnetic induction were the following: (1) electromagnetic induction phenomenon, (2) conditions to generate induced current, (3) Lenz’s law, (4) the right-hand rule, and (5) Faraday’s law. These concepts and laws comprise the first dimension of attributes (i.e., knowledge content).

Based on Benjamin Bloom’s research, Anderson et al. (2001) divided learning results in the cognitive domain into several progressive levels based on explicit behaviors that correspond to the degree to which students understand the subject. The learning results in the cognitive domain were divided into six levels: knowledge, comprehension, application, analysis, synthesis, and evaluation. In line with this, Lee et al. (2011) divided the cognitive dimensions measured in the math items of Trends in Mathematics and Science Study (TIMSS) test into three levels: knowing, applying, and reasoning. In the present study, the five experts determined that the relevant questions could be divided into four levels according to the learning results of the examination: (1) knowledge, (2) understanding, (3) application, and (4) integrated application.

##### Knowledge

If students were able to correctly answer questions that examine concepts, content of laws, and corresponding physical phenomena, their learning results were defined as “knowledge.” For example, the following question examines the attribute “electromagnetic induction phenomenon: knowledge.”

*Which of the following phenomena is electromagnetic induction?*

*A. A current is subjected to a force in a magnetic field.*

*B. There is a magnetic field around the current.*

*C. A soft iron bar can be magnetized by a magnetic field.*

*D. The changing magnetic field causes an electric current to be generated in a closed conductor.*

##### Understanding

If students were able to correctly answer questions that use laws for calculation and reasoning, their learning results were defined as “understanding.” For example, the following question examines the attribute “conditions to generate induced current: understanding.”

*Which situation can induce current?*

*A. The conductor moves in a cutting magnetic field line.*

*B. A part of the closed circuit moves parallel to the magnetic field.*

*C. A part of the closed circuit cuts the magnetic field lines in a magnetic field.*

*D. None of this is true.*

##### Application

If students were able to correctly answer questions regarding two-related physical processes and two-related physical objects which do not involve knowledge beyond electromagnetism, their learning results were defined as “application.” For example, the following question examines the attribute “Lenz’s law: application.”

**Figure 2:**
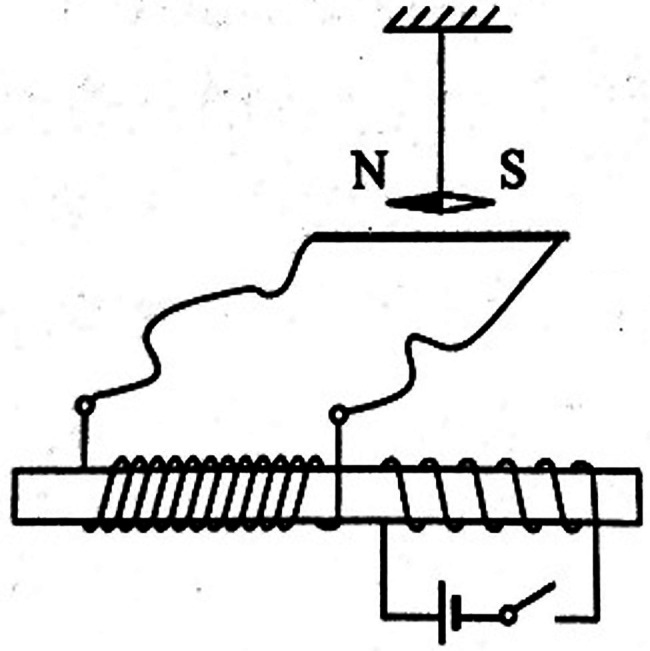
The circuit of example question that examines the attribute “Lenz’s law: application.”

*As shown in the Figure 2, two coils are wound around an iron core. One coil is connected to the switch and the power supply, and the other coil is connected in a loop with a straight wire placed horizontally in a north–south direction. A magnetic needle is suspended directly over the straight wire and stands still when the switch is off. Which choice is true?*

*A. At the instant after the switch is on, the N pole of the needle points inward to the paper.*

*B. After the switch is on and held for a period of time, the N pole of the magnetic needle points inward to the paper.*

*C. After the switch is on and held for a period of time, the N pole of the magnetic needle points out of the paper.*

*D. When the switch is closed for a period of time and then opened, the N pole of the magnetic needle points outward to the paper.*

##### Integrated Application

If students were able to correctly answer questions regarding more than two-related physical processes and more than two-related physical objects which involve knowledge beyond electromagnetism, their learning results were defined as “integrated application.” For example, the following question examines the attribute “Faraday’s law: integrated application.”

**Figure 3:**
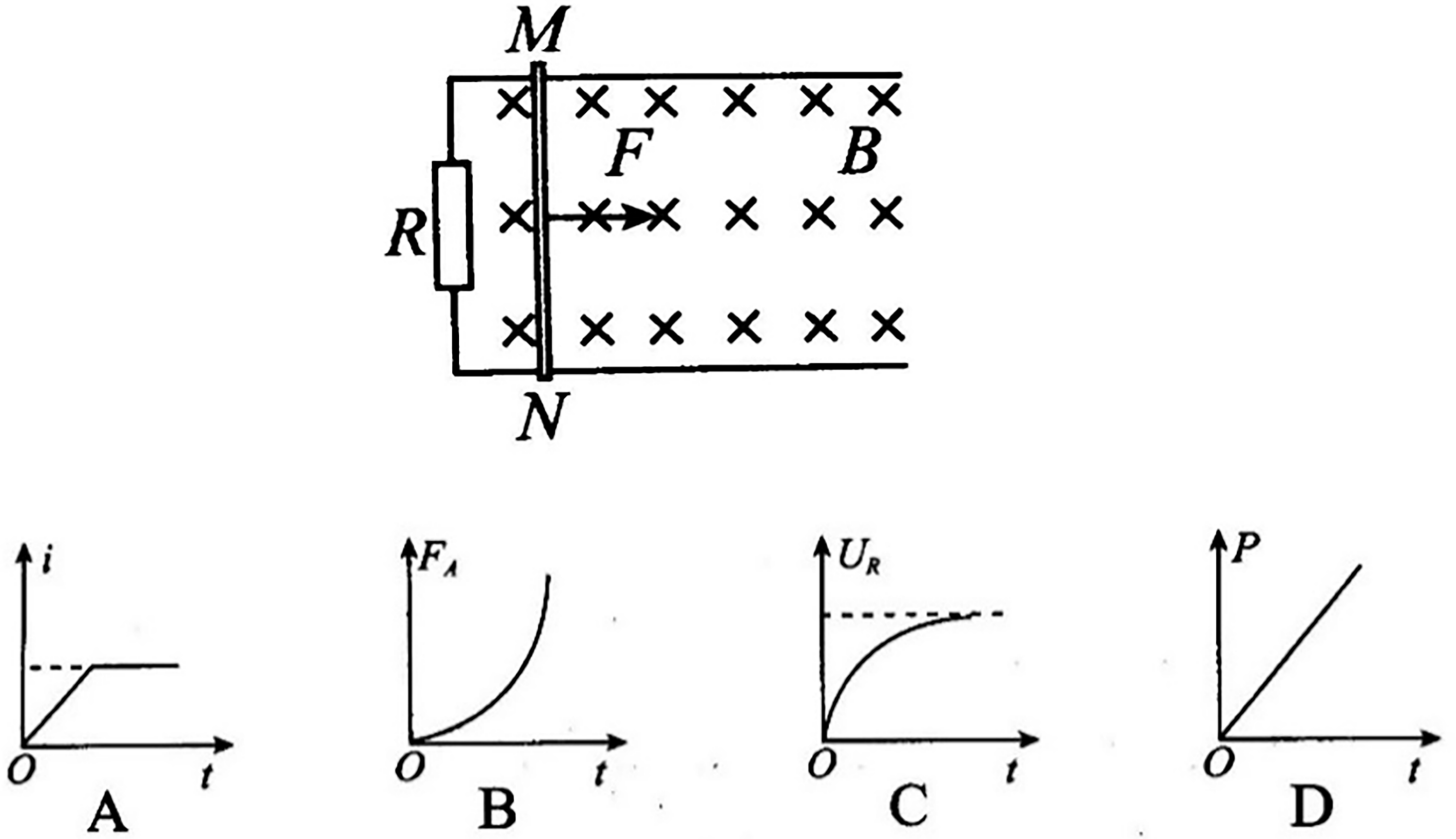
The circuit and options of example question that examines the attribute “Faraday’s law: integrated application.”

*As shown in the Figure 3, the smooth parallel metal with no resistance and spacing of L is horizontally placed in the uniform magnetic field with a magnetic induction intensity of B and direction of vertical downward, and the left end of the rail is connected with a resistance R. The metal bar MN with mass m and resistance R is placed on the rail and moves from rest under the action of the horizontal external force F perpendicular to the metal bar. The relationship between the F and the speed v of the metal bar is* $F=F_{0}+kv$ *(*$F_{0}$ *and k are constant). The metal bar and the rail are always vertical and in good contact. The induced current in the metal bar is i, the ampere force is* $F_{A}$*, the voltage at both ends of the resistance R is* $U_{R}$*, and the power of the induced current is P. Which graph might correctly represent the trend of physical quantities over time?* (The options are shown in Figure 3).

On average, the consistency among the five experts for the knowledge content and learning result of each question was 83.0%. Based on the cognitive attributes marked by the experts, if three or more experts agreed on the level of learning results examined by the item, they were included as cognitive attributes. Ultimately, 12 attributes were determined, which are shown in Table 1.

**Table 1:** Multi-level attributes.

Attributes	Detailed description
EIP	Electromagnetic induction phenomenon: Knowledge
CIC1	Conditions to generate induced current: Knowledge
CIC2	Conditions to generate induced current: Understanding
LL1	Lenz’s law: Knowledge
LL2	Lenz’s law: Understanding
LL3	Lenz’s law: Application
RHR1	Right-hand rule: Knowledge
RHR2	Right-hand rule: Understanding
FL1	Faraday’s law: Knowledge
FL2	Faraday’s law: Understanding
FL3	Faraday’s law: Application
FL4	Faraday’s law: Integrated application

Concepts or rules that were not within the examination scope of college entrance exams and academic achievement tests (i.e., higher levels) were considered beyond the scope of this study and were thereby not included.

#### Preparing the Test

A Q-matrix which conforms to the goal of cognitive diagnostic was developed prior to compiling the cognitive diagnostic test. Based on the principle that each attribute must be tested at least thrice, the Q-matrix was developed for two sets of parallel tests (Supplementary Table S1). Using the cognitive attributes and question bank items, two sets of 36 items which follow the measurement patterns were selected from the question bank for Form A (pre-test) and Form B (post-test): Electromagnetic Induction Cognitive Diagnostic Test (EICD). In the EICD test, the items that examined the attributes of “knowledge” were fill-in-the-blank questions; the other items were multiple-choice questions.

To test the EICD’s quality, 680 senior high school students were recruited by convenience sampling to participate in the initial test: 442 answered Form A and 238 answered Form B. Before the test, the students and their guardians provided written informed consent for the study. The students were told that participation was voluntary and that they were allowed to withdraw at any time. In addition, participants were also told that the tests must be accomplished independently and that their results will not be part of their physics class evaluation.

The overall quality of test and items was judged according to Classical Measurement Theory (CTT). The reliability of the EICD test (initial) was measured by the CTT-based Cronbach’s α coefficient. The CTT score is calculated as 2 points for correct answers at the “knowledge” level items, 3 points to the “understanding” level items, 4 points to the “application” level items, and 5 points for the “integrated application” level items. The α coefficients of EICD test (initial) Forms A and B were 0.7634 and 0.7364, respectively. Except for Item 3 and Item 11, the difficulty coefficient of the items was basically consistent with the learning level dimension of the attributes examined by the item. The difficulty coefficients ranged from [0.17, 0.97]. Item 3 and Item 11 were both “knowledge” level items, but the difficulty coefficients indicate that they were too difficult. Except for Item 11, the discrimination of items is between [0.21, 1.0]. Items with a difficulty coefficient above 0.4 accounted for more than half of the total items. Item 3 and Item 11 were replaced by items of the same level of assessment in the item bank. So far, after the initial test, the quality of the EICD test had been optimized, and the EICD test (formal) had been developed, with 36 items for each of the Form A and Form B, which are used for the formal test.

#### Formal Test

Because CDA requires a sufficient number of participants, approximately 1,000 responses were needed prior to form the response matrix with the test of the teaching experiment. In this section, the method for diagnostic classification will be discussed.

##### Participants

Using stratified sampling, 1,615 senior high school students from seven high schools in Eastern, Northwest, Southwest, and Central China participated in the formal cognitive diagnostic test, which was conducted in June and July 2019. In total, 861 participants effectively completed Form A, while 849 participants effectively completed Form B. Of these, 95 participants completed both Form A and B. Participants were given 1 h to complete the test. Written informed consent was provided by the participants and their guardians prior to the study.

##### Data Analysis

The quality of the EICD test(formal) was measured by CTT score. To examine whether the EICD test (formal) can reflect the real learning situation of students, 267 participants that completed the same Academic Level Test (2019 High School Academic Level Test in Yunnan Province) were selected. The grades of the Academic Level Test were A, B, and C from high to low. Correlations between Academic Level Test grades and EICD average scores were compared.

In order to explore whether Form A and B meet the requirements of parallel papers, the mean (*M*), standard deviation (SD), item difficulty (*P*), and item discrimination (*D*) of students’ scores in Form A and B were compared.

##### Model Selection

Selecting an appropriate CDM was a necessary condition for obtaining reasonable diagnostic feedback. In this study, because the attributes correspond to specific mastery levels, students were considered to have mastered an attribute (attaining a commanded mastery level) if the items were answered correctly. The hierarchical structure between attributes was not considered. Therefore, the DINA model and the GDM model, which were both non-compensatory and also do not consider hierarchical structure, were selected as alternative models in this study. The relative fitting parameters showed that the theoretical relative fitting degree of GDM model is the better fit (AIC = 32673.6, BIC = 33392.1), than DINA model (AIC = 39938.5, BIC = 59765.5). During the formal test, when assessed with the DINA and GDM models, it was found that the same participant can have different patterns of attribute mastery. When participants with different diagnostic results were interviewed about the answered items and when the attribute lists were compared, the diagnostic accuracy rate of the DINA model appeared to be higher than that of the GDM model (Supplementary Table S2). Cai et al. (2013) used the method of data simulation to compare the diagnostic accuracy of the five commonly used models for mastering patterns in different situations. The results showed that under any knowledge state distribution, with a large sample size of about 1,000, and in the case of a large number of cognitive attributes, the accuracy of the DINA model diagnosis is relatively good. Hence, the present study adopted the DINA model for CDA. The DINA model was first proposed by Macready and Mitchell (1977) and was subsequently improved by Haertel (1989). Currently, it has now become a comparative, basic, and commonly used model in research (Junker and Klaas, 2001; Templin and Henson, 2006). The answer matrix was analyzed on the flexCDMs.^1^

The cognitive diagnosis reliability of the 12 attributes was measured by the consistency index of the attribute test–retest reliability. Assuming that the students’ mastery probability of attributes remains unchanged, the two-by-two contingency tables on the correlation of the mastery of attributes in the test and retest may be obtained and used as the index of attribute reliability in the cognitive diagnostic test (Templin and Laine, 2013).

##### Diagnostic Classification Method

**Figure 4:**
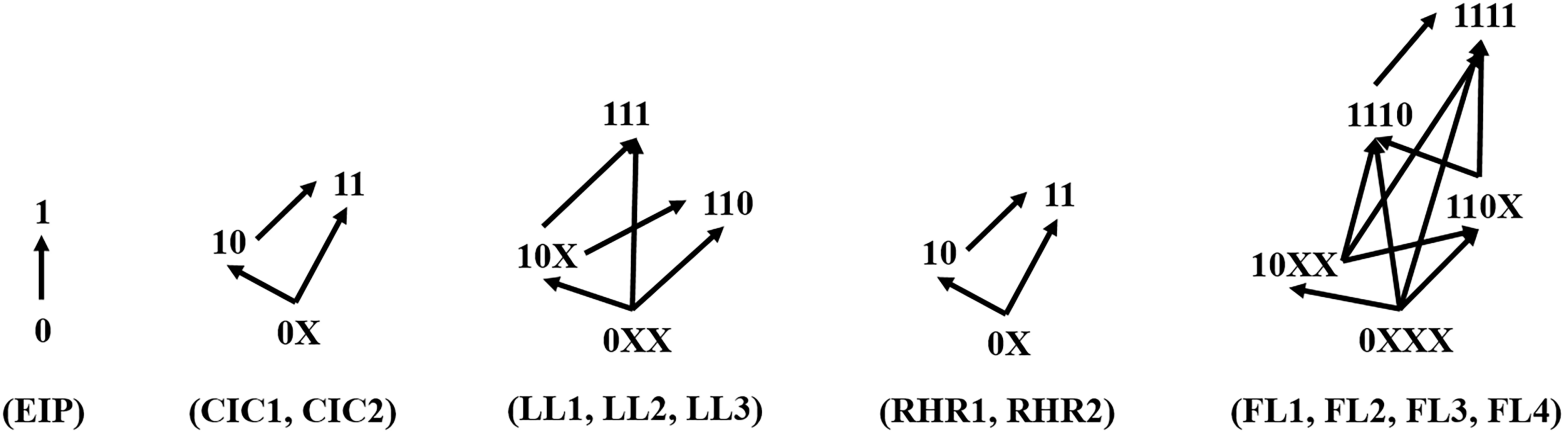
Classification schemes and learning paths based on attribute mastery pattern. The 12 attributes are divided into five groups according to the knowledge content. Students are categorized by their mastery pattern for each attribute group. 1 represents mastered the attribute, 0 represents not mastered the attribute, and X represents 1 or 0. The arrow represents the learning path after diagnosis and classification, the tail of the arrow represents the attribute mastery pattern of the pre-test, and the tip refers to the target attribute mastery pattern after the layered remedial teaching.

The DINA model gives feedback on the mastery pattern of each student for the attributes. The mastery pattern of a student is a vector composed of 12 elements that equal 0 (attribute not mastered) or 1 (mastered attribute). In this study, the vector of mastery pattern was divided into five small mastery pattern vectors according to the five knowledge contents: (EIP), (CIC1 and CIC2), (LL1, LL2, and LL3), (RHR1 and RHR2), (FL1, FL2, FL3, and FL4). (See the notes to Table 1 for the full meaning of the acronyms). The five small mastery patterns were classified, as shown in Figure 4. The purpose of the diagnostic classification during the pre-test (Form A) was to identify the students’ level of learning regarding the five knowledge contents of electromagnetic induction. Different mastery patterns of the attributes imply different learning levels. Then, according to the results of diagnosis and classification, multi-level remedial teaching can be carried out.

The purpose of post-test diagnostic classification was to test whether the student has achieved the target mastery pattern. Figure 4 shows the method of diagnostic classification and learning paths for multi-level remedial teaching, with X representing either 0 or 1. Each student was classified five times according to the attribute mastery pattern of the five knowledge contents. In a knowledge content mastery pattern, students are classified according to the lowest level attributes that they have not mastered, and students with the same minimum level attributes are classified into one category. The end of the arrow is the diagnostic classification result of the attribute mastery pattern in the pre-test, and the tip of the arrow represents the target attribute mastery pattern in the post-test.

### Multi-level Remedial Teaching Design

Before designing a multi-level remedial teaching plan, it is necessary to understand the cognitive errors of students at each mastery pattern classification. According to the diagnosis classification method, each student in the experimental group was classified into five groups in five knowledge contents. As shown in Figure 2, there were 17 groups in total. If a student was classified in groups without a 0 element, they were no longer included for remedial teaching. There was a total of 12 mastery pattern classifications in which corresponding cognitive errors had to be determined. In order to obtain the cognitive errors of students under each classification, three students were selected from each group for a total of 36 students. The 36 students were then asked to restate their thoughts when they completed either form (A or B). The researchers recorded and analyzed the cognitive errors, and then summarized the common characteristics of the cognitive errors made at each classification. Afterward, remedial teaching schemes were then designed with the aim of addressing these cognitive errors.

#### Think-Aloud Protocols

Before the think-aloud protocols, the 36 students were informed by their physics teacher that this was a teaching research in which students would be asked to repeat their thoughts during the test, that participation was voluntary, and that they could quit at any time during the process. The students were then asked to go into a classroom one-by-one and to repeat their thoughts aloud regarding Form A or Form B. The three researchers then noted the participants’ cognitive errors. Finally, the three researchers discussed the common cognitive errors made by the students, as shown in Table 2.

**Table 2:** The cognitive errors of each mastery pattern classification.

Mastery pattern	No.	Cognitive errors
EIP = 0	1	The interaction of electricity and magnetism was incorrectly considered as electromagnetic induction
	2	Confusion over the physical principles on which generators and motors are based
(CIC1, CIC2) = (0X)	3	Misunderstanding that magnetic flux changes if the conductor cuts the magnetic field lines
(CIC1, CIC2) = (10)	4	Misunderstanding that an induced current can be generated if the conductor cuts the magnetic field lines
	5	Misunderstanding that the unclosed coil has no magnetic flux, and the magnetic flux changes when it is closed again
	6	Did not understand the magnetic field distribution around the bar magnet
(LL1, LL2, LL3) = (0XX)	7	Did not understand the concept of flux change
	8	Could not describe Lenz’s law
(LL1, LL2, LL3) = (10X)	9	Could not translate a change in the physical situation into a change in the magnetic flux, thereby not knowing how to use Lenz’s law
	10	Lenz’s law was not understood in terms of energy, and the transformation of functional relations in electromagnetic induction was not understood.
	11	In the process of using the formula, the formula was mistaken for Lenz’s law
(LL1, LL2, LL3) = (110)	12	In physical situations, the hindrances of Lenz’s law were not used to determine the direction of the induced current
(RHR1, RHR2) = (0X)	13	Students could not distinguish between the right-hand rule, left-hand rule, and ampere rule
	14	Misunderstanding that the direction of the conductor cutting magnetic field lines is the direction of the conductor force
	15	It was incorrectly believed that the direction of the induced electromotive force is opposite to that of the induced current
(RHR1, RHR2) = (10)	16	Incorrectly used the left-hand rule to solve the electromagnetic induction problem
	17	Did not understand the direction of the current or magnetic field lines in the diagram
(FL1, FL2, FL3, FL4) = (0XXX)	18	Did not know the basic concept of Faraday’s law
	19	Could write Faraday’s law formula but could not explain Faraday’s law
	20	Inability to distinguish between the rate of change and quantity of change
(FL1, FL2, FL3, FL4) = (10XX)	21	The left-hand rule was used
	22	Did not understand the circuit knowledge. Misjudged the direction of current and potential inside the source
	23	Did not know that the conductor cutting the magnetic field lines is equivalent to the power supply
(FL1, FL2, FL3, FL4) = (110X)	24	Did not know that the conductor cutting the magnetic field lines is equivalent to the power supply
	25	Did not know the terminal voltage
	26	Did not know that the uniform increase of *B* and *δ B/δ t* have the same meaning, and mistook the former for the magnetic flux uniform increase
(FL1, FL2, FL3, FL4) = (1110)	27	Could not find the functional relationship between each physical quantity and *t*
	28	No in-depth understanding of the concept of acceleration
	29	Did not consider that the width of the field is larger than the edge of the wire
	30	Calculation error

*EIP, Electromagnetic induction phenomenon: knowledge; CIC1, Conditions to generate induced current: knowledge; CIC2, Conditions to generate induced current: understanding; LL1, Lenz’s Law: knowledge; LL2, Lenz’s Law: understanding; LL3, Lenz’s Law: application; RHR1, Right-hand rule: knowledge; RHR2, Right-hand rule: understanding; FL1, Faraday’s law: knowledge; FL2, Faraday’s law: understanding; FL3, Faraday’s law: application; and FL4, Faraday’s law: integrated application*.

#### Multi-level Remedial Teaching Scheme

As Table 2 shows, students whose attribute mastery pattern was (EIP) = 0, (CIC1, CIC2) = (0X), (LL1, LL2, LL3) = (0XX), (RHR1, RHR2) = (0X), (FL1, FL2, FL3, FL4) = (0XXX) made errors in identifying related phenomena and concepts, repeating laws, and comprehending the essence of laws. Therefore, the remedial teaching scheme for these attribute mastery patterns focused on experiment and discussion. For students, whose attribute mastery pattern was (CIC1, CIC2) = (10), (LL1, LL2, LL3) = (10X), (RHR1, RHR2) = (10), (FL1, FL2, FL3, FL4) = (10XX), their cognitive errors involved the misuse of laws and not understanding the essence of the law. Therefore, the remedial teaching scheme for these attribute mastery patterns focused on experiment, speculation, and discussion. Lastly, for students whose attribute mastery pattern was (LL1, LL2, LL3) = (110), (FL1, FL2, FL3, FL4) = (110X) or (111X), their cognitive error involved not finding the relationship between multiple physical processes and quantities in the physical situation, or lacking a deep knowledge beyond the topic of electromagnetic induction. Therefore, the remedial teaching scheme for these levels of classification included guiding students in discussing the aforementioned topics together. Due to manuscript limitations, only three different levels of remedial teaching schemes are listed below. If readers are interested, the authors may be contacted for all of the multi-level remedial teaching schemes.

(a) EIP = 0 level remedial teaching scheme

Based on the results of the think-aloud protocols, multi-level remedial teaching should not only make students aware of electromagnetic induction, but it should also allow them to correctly distinguish electromagnetic induction from other electromagnetic phenomena. Based on this, the following remedial teaching plan was designed as:

[Teaching method] Experiment and discussion.

[Teaching aim] To be able to distinguish three types of electromagnetic interactions.

[Experimental material] A core, two solenoids, a magnetic needle, a DC power supply, a switch, a slide rheostat, wires, a sensitive galvanometer, and a bar magnet.

**Figure 5:**
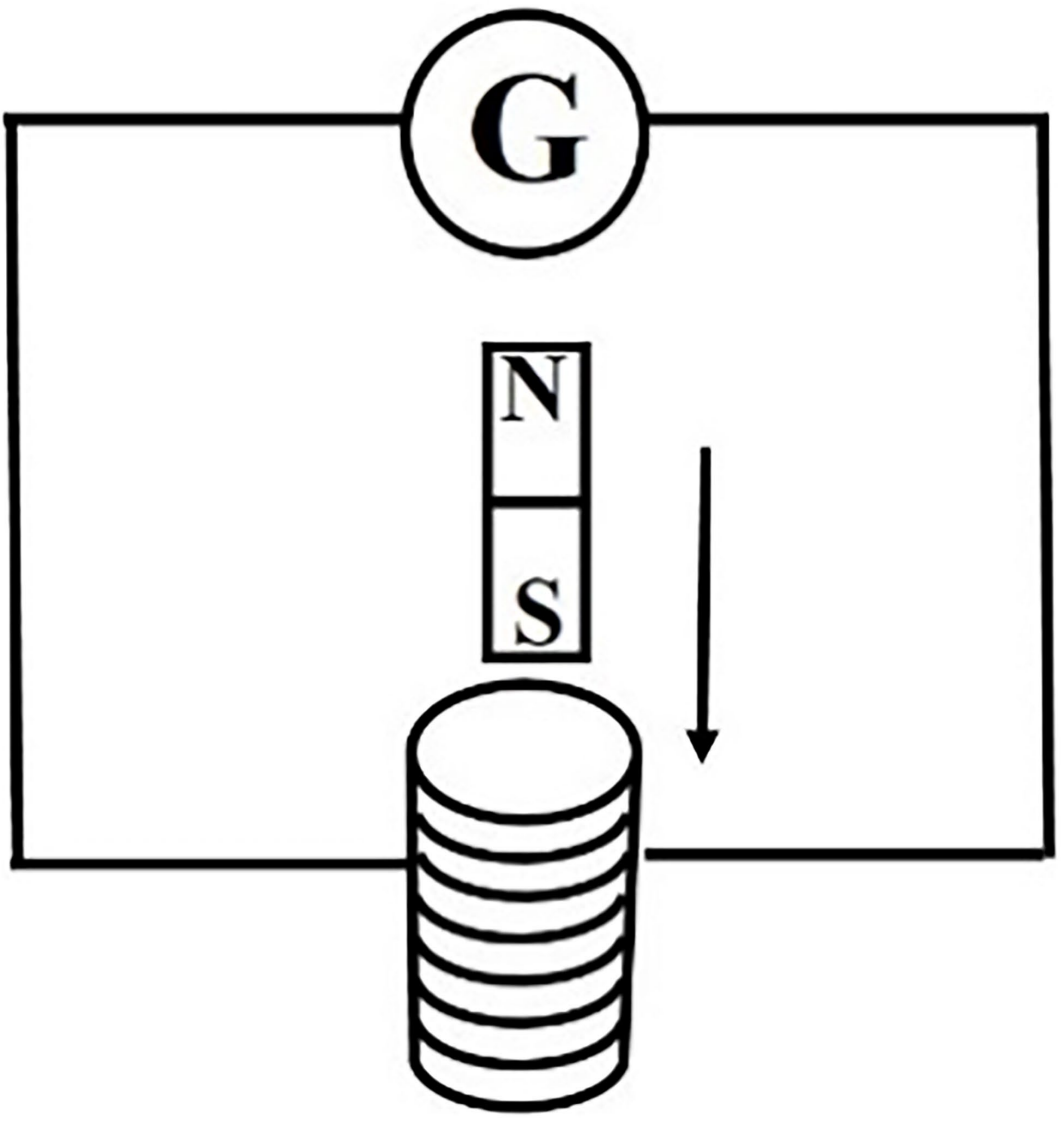
Experimental circuit i of EIP = 0 level remedial teaching scheme.

[Experimental circuit i is shown in the Figure 5]

[Discussion topic i] Corresponding to cognitive error 1 in Table 2: *Is there a power supply in the circuit? How does the pointer of the sensitive galvanometer deflect at the moment when the bar magnet enters and leaves the solenoid? Why is the pointer of the sensitive galvanometer deflected? What phenomenon does this process belong to?*

**Figure 6:**
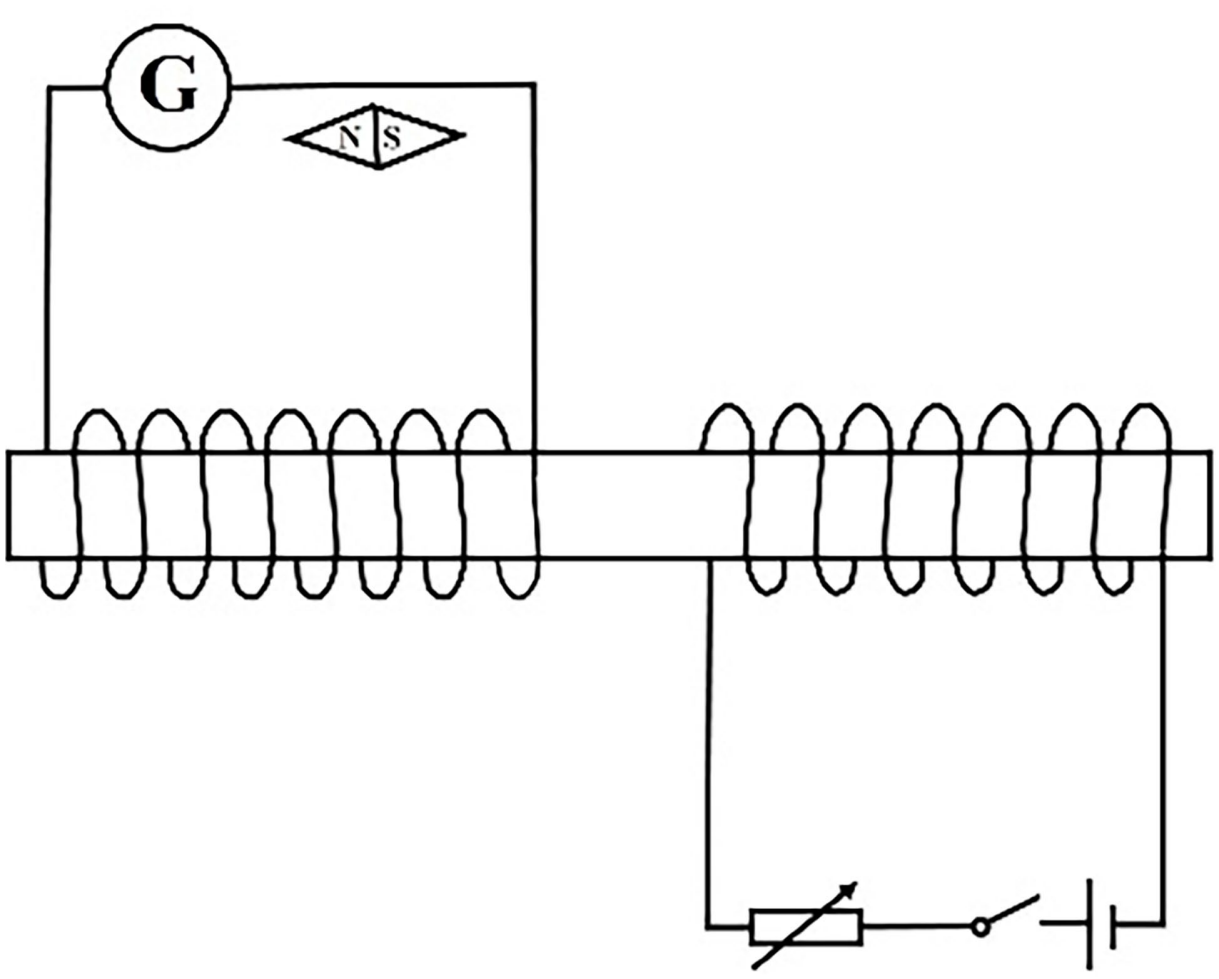
Experimental circuit ii of EIP = 0 level remedial teaching scheme.

[Experimental circuit ii is shown in the Figure 6]

[Discussion topic ii] Corresponding to cognitive error 1 in Table 2: *Observe the change of the magnetic needle as the switch closes and opens. What kind of electromagnetic interaction is involved in each link of the experiment?*

[Discussion topic iii] Corresponding to cognitive error 2 in Table 2: *Discuss the difference and connection between the generator and motor.*

(b) (LL1, LL2) = (10) level remedial teaching scheme

According to the results of the think-aloud protocols, the following remedial teaching plans were designed to address the cognitive errors of students in order to reach the attribute mastery pattern of (LL1, LL2) = (11):

[Teaching method] Experiment, speculation, and discussion.

[Teaching aim] To use Lenz’s law to determine the direction of the induced current in a simple situation.

[Experimental material] A bar magnet and a Lenz’s law demonstrator.

**Figure 7:**
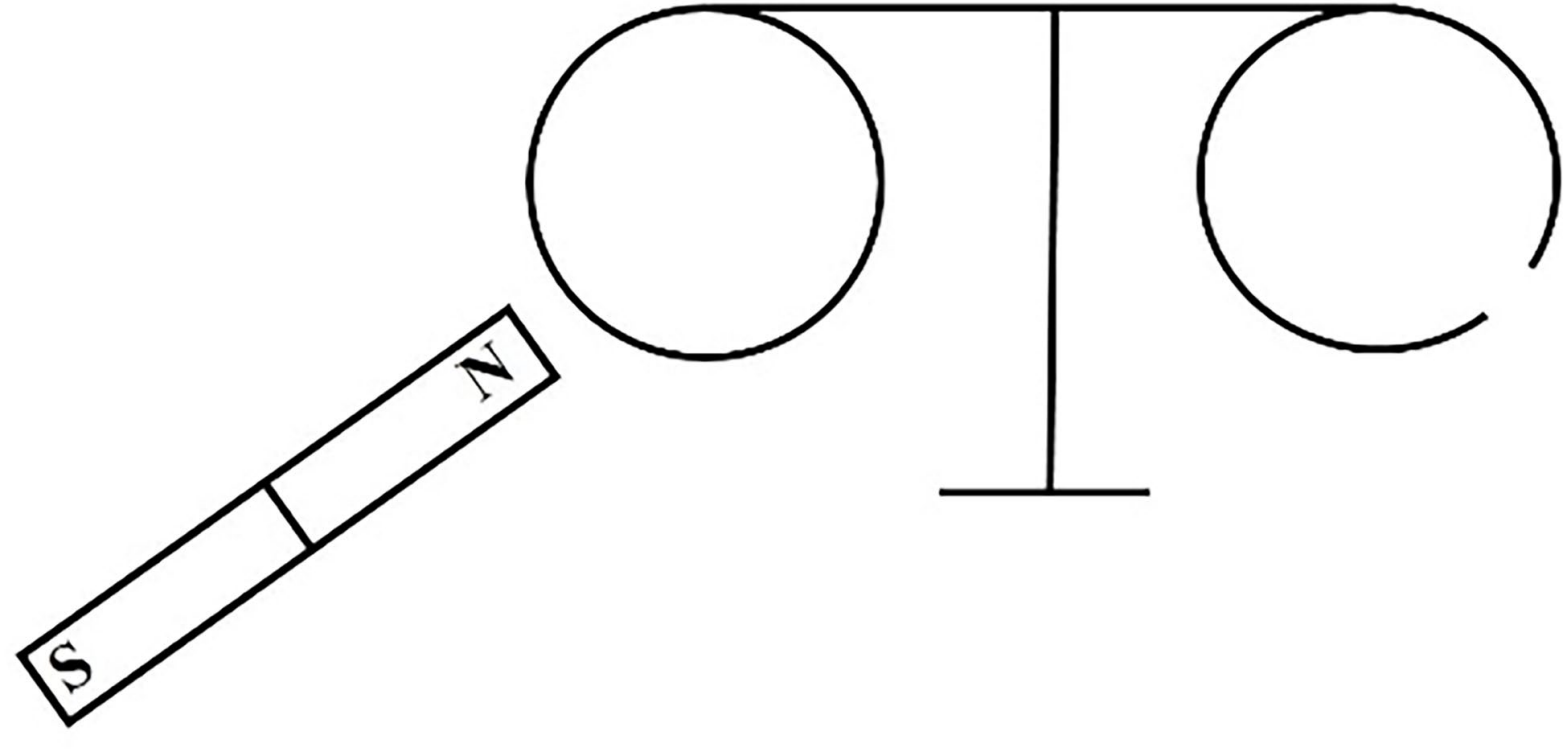
Experimental circuit i of (LL1, LL2) = (10) level remedial teaching scheme.

[Experimental circuit i is shown in the Figure 7]

[Discussion topic i] *Enter and leave the two aluminum rings in a bar magnet; which will generate the electromagnetic induction and why?*

[Teaching aim i] Corresponding to cognitive error 9 in Table 2: To enable students to judge the change of magnetic flux in a closed loop in a physical situation, so as to judge the direction of the induced current.

**Figure 8:**
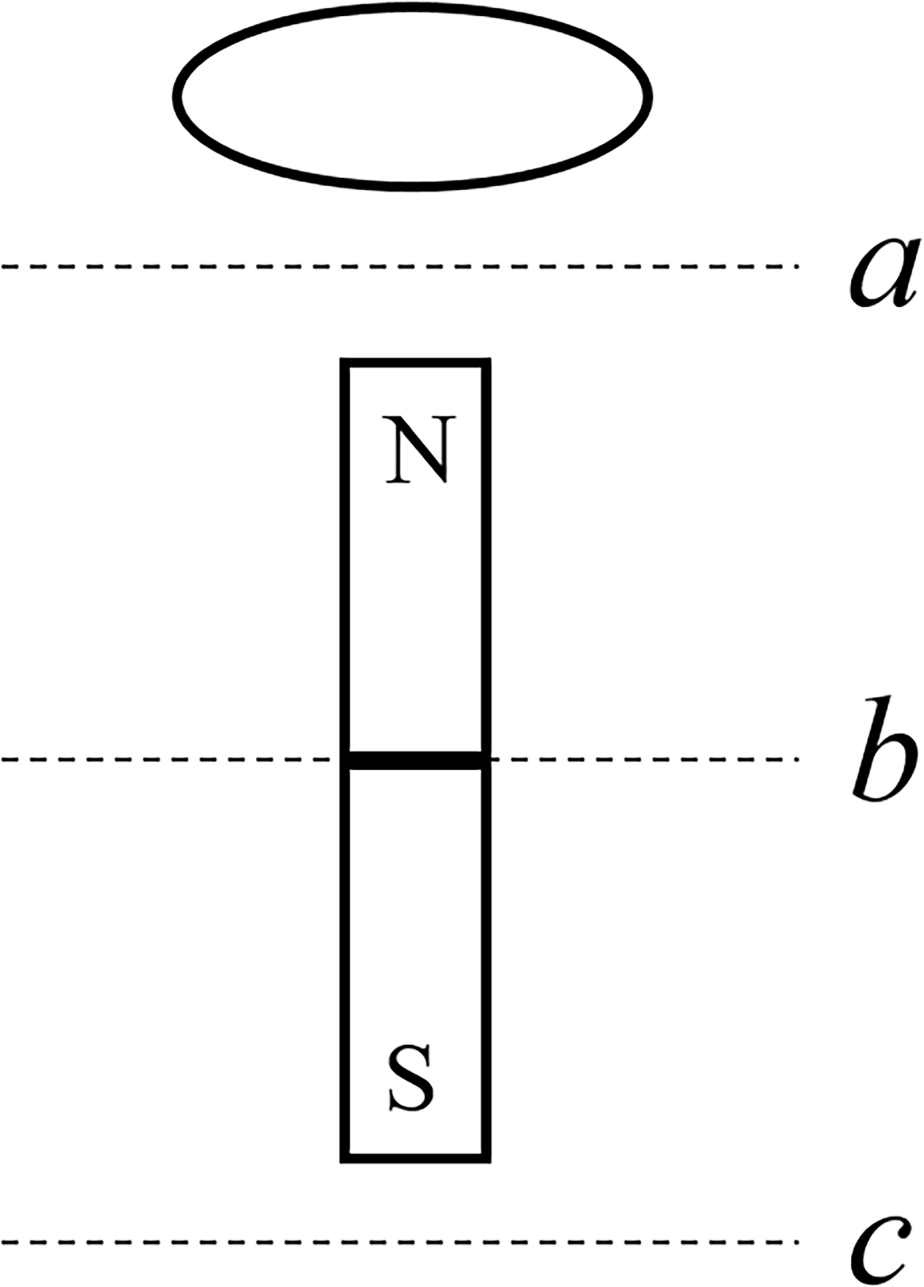
The physical situation circuit of (LL1, LL2) = (10) level remedial teaching scheme.

[Speculation ii : The physical situation is shown in Figure 8]

[Discussion topic ii] *Imagine the physical process of a speculative drawing shown in Figure 8. Hold the bar magnet and let the coil move. The induced current directions of the coil at positions a, b, and c are discussed. Is the force exerted on the coil at a and c by the bar magnet dynamic or resistance? Is the work done by the force of gravity on the coil fully converted to kinetic energy as if it were in free fall?*

[Teaching aim ii] Corresponding to cognitive error 10 in Table 2: To clarify the relationship between work and energy in electromagnetic induction.

[Discussion topic iii] *What is the fundamental reason for the use of the formula of “increase, reverse, decrease, same” and “come, go, refuse, and stay”?*

[Teaching aim iii] Corresponding to cognitive error 11 in Table 2: To develop an in-depth understanding of Lenz’s law behind the formula.

(c) (LL1, LL2, LL3) = (110), (FL1, FL2, FL3, FL4) = (110X) or (1110) levels remedial teaching scheme

The results of the think-aloud protocols showed that students usually fail to reach the “application” and “integrated application” level when they lack further physics knowledge and are unable to correctly relate two or more physical processes and laws. For these students, the remedial teaching method involved the teaching assistant leading the students in a discussion of the corresponding problems in classifications so that the students may better understand the connection between the physical processes from the analysis of the physical processes and the applied physical laws. With this, long-term remedial teaching should strengthen the application of additional physical knowledge. To improve the learning of the students, the remedial teaching plan was designed as follows:

[Teaching method] Discussion.

[Teaching material] Questions at the corresponding level in Form A.

[Discussion topic] Each student explains the reasons for their choice through a speech, other students give their opinions, and one student summarizes the results of the discussion.

[Teaching assistant (TA) guidance] The TA guides the students in analyzing the physical process involved in the questions and the physical laws corresponding to the physical process. Then, they find a physical quantity that connects multiple physical processes.

### Implementing Multi-level Remedial Teaching and Post-test

#### Participants

Among the seven schools that participated in the formal test, the authors selected three schools of different levels from Ministry of Education evaluations in the same city to conduct the experiment. School 1 (S1) was a private high school belonging to the lowest level in the local area; school 2 (S2) was a general public high school belonging to the middle level in the local area; and school 3 (S3) was a Level 1 high school, which is considered a high-level high school in the local area. In each school, two parallel classes were selected as the experimental group and control group. Experimental groups S1, S2, and S3 had 30, 52, and 48 participants, respectively, while the matching control groups had 29, 50, and 49 participants, respectively.

#### Multi-level Remedial Teaching Process

The students of the experimental groups and control groups were given 1 h to complete Form A. For the response matrix, the results of the experimental and control groups were then combined with those of the students who participated in the formal test of Form A. The DINA model was then used for cognitive diagnosis, and the experimental group was classified according to the method in stated in the “Diagnostic classification method” section. In the experimental group, researchers and teaching assistants completed multi-level remedial teaching according to the classification results, and each attribute mastery pattern classification group spent approximately 30 min learning. Each student in the experimental group could be classified into multiple groups and could receive remedial teaching for up to 2.5 h. In the control group, in order to minimize the influence of the number of students on the teaching effect, the students in the control group were randomly divided into a group of approximately 10 students and received approximately 2.5 h of instruction on the correct answers and solutions to the Form A questions. Table 3 shows the process of the multi-level remedial teaching experiment.

**Table 3:** The process of the multi-level remedial teaching experiment.

Day-period	Experimental group				Control group			
Day1	Pre-test (Form A)							
Day2	There is no task for students, while researchers complete the classification							
	Classification group/remedial teaching	S1(N)	S2(N)	S3(N)	Remedial teaching method	S1(N)	S2(N)	S3(N)
Day3-1	EIP = 0	22	38	45	Explain the correct answers in Form A	8	13	13
Day3-2	(CIC1, CIC2) = (0X)	21	23	28				
Day3-3	(CIC1, CIC2) = (10)	3	29	22				
Day4-1	(LL1, LL2, LL3) = (0XX)	19	29	28	Explain the correct answers in Form A	7	13	12
Day4-2	(LL1, LL2, LL3) = (10X)	5	45	48				
Day4-3	(LL1, LL2, LL3) = (110)	6	8	7				
Day5-1	(RHR1, RHR2) = (0X)	17	11	3	Explain the correct answers in Form A	7	12	12
Day5-2	(RHR1, RHR2) = (10)	10	22	25				
Day5-3	(FL1, FL2, FL3, FL4) = (0XXX)	22	17	20				
Day6-1	(FL1, FL2, FL3, FL4) = (10XX)	5	26	24	Explain the correct answers in Form A	7	12	12
Day6-2	(FL1, FL2, FL3, FL4) = (110X)	2	18	6				
Day6-3	(FL1, FL2, FL3, FL4) = (1110)	0	7	20				
Day7	Post-test (Form B)							

#### Post-test

After remedial teaching was completed, students in both control and experimental groups spent 1 h simultaneously completing Form B. The response results were then combined with those of the students who participated in the formal test of Form B into the response matrix.

#### Data Analysis

The DINA model was used for cognitive diagnosis in post-test. We focused on whether students of each pre-test-based classification achieved the target attribute mastery pattern in the post-test, following the path in Figure 4. To test the effect of multi-level remedial teaching, the researchers defined a target attribute mastery pattern achievement rate index to determine whether post-test targets were reached. This rate was calculated as follows:


(1) $$\eta_{i}=\frac{n_{i}}{N_{i}},$$

where $\eta_{i}$ is the achievement rate of the No. *i* attributes mastery pattern classification; $N_{i}$ is the number of students in the No. *i* attributes mastery pattern classification in the pre-test; and $n_{i}$ is the number of students who had achieved the target attribute mastery pattern in the post-test and were in the No. *i* attributes mastery pattern classification in pre-test.

To compare the differences in remedial teaching among the three schools, ANOVA was performed with the method of remedial teaching as the independent variable, while the students’ target attribute mastery pattern achievement rate was considered as the dependent variable. Microsoft Excel 2016 was used to perform a single factor ANOVA.

## Results

### Assessment and Classification

#### Quality of the EICD Test

**Figure 9:**
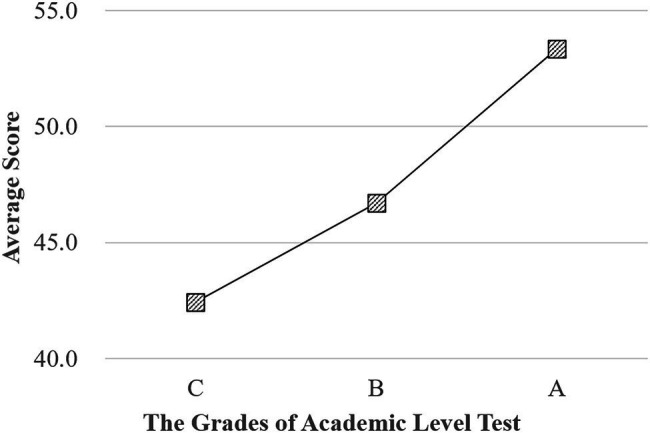
The relationship between the EICD test CTT scores and the grades of the Academic Level Test. The ordinate is the average score in the EICD test(formal) of the students who obtained the A, B, or C grade.

Figure 9 shows the relationship between the EICD test CTT scores of the participants and the grades of the Academic Level Test. The grades of the Academic Level Test are A, B, and C from high to low, which are marked on the abscissa of Figure 9. The ordinate is the average score in the EICD test(formal) of the students who obtained the A, B, or C grade. The average score of the EICD (formal) test is basically linear and positively related to the Academic Level Test grades, indicating that the EICD test(formal) can reflect the real learning situation of students.

In order to explore whether Form A and B meet the requirements of parallel papers, Table 4 presents the mean (*M*), standard deviation (SD), item difficulty (*P*), and item discrimination (*D*) of students’ scores in Form A and B. It was found that the descriptive statistical indicators of the two Forms were basically the same. In addition, the one-way ANOVA with the test paper as the independent variable and the score as the dependent variable showed (*p* = 0.286) that the difference of the test paper had no significant effect on the total score. Finally, because the two Forms measured the same knowledge contents and learning level, containing the same number of questions, question types, and the same test Q-matrix, the Form A and Form B in this study can be considered to meet the requirements of parallel tests.

**Table 4:** The statistical description results of Form A and B.

	*M* + SD	*P*	*D*
Form A	59.3 + 18.3	0.58	0.48
Form B	61.3 + 21.9	0.60	0.48

*M*, *Mean; SD, Standard deviation; P*, *difficulty; and D*, *discrimination.*

The cognitive diagnostic reliability of the 12 cognitive attributes of Forms A and B is shown in Table 5. The cognitive diagnostic reliability of all cognitive attributes is above 0.9, which indicates that the reliability of cognitive attributes was sufficient and that the test truly reflects the learning results.

**Table 5:** The reliability of cognitive attributes.

	EIP	CIC1	CIC2	FL1	FL2	FL3	RHR1	RH2	FL1	FL2	FL3	FL4	Mean
Form A	0.9766	0.9847	1	0.9797	1	0.9829	1	0.9908	0.9972	0.9961	1	1	0.9923
Form B	0.9714	0.9649	0.9311	0.9369	1	0.9482	0.9734	0.9624	0.9896	0.9772	0.9812	0.9851	0.9684

### Classification Results

The students participating in the formal test were classified based on the classification method in the Figure 4. The classification results are shown in Table 6.

**Table 6:** Classification results.

Attributes group	Attribute mastery pattern	Proportion
EI	0	0.3082
	1	0.6918
CIC1, CIC2	0X	0.2509
	10	0.3526
	11	0.3965
LL1, LL2, LL3	0XX	0.2772
	10X	0.2374
	110	0.1275
	111	0.3579
RHR, RHR2	0X	0.2076
	10	0.1906
	11	0.6018
FL1, FL2, FL3, FL4	0XXX	0.2509
	10XX	0.1380
	110X	0.2146
	1111	0.0702
	1111	0.3263

Table 6 shows the classification result of the formal cognitive diagnostic tests. The first column is the attributes group. The vectors in the second column represent the attribute mastery patterns at the same group of knowledge contents. X means 0 or 1. The data in the third column are the proportion of the classified population. EIP, Electromagnetic induction phenomenon: knowledge; CIC1, Conditions to generate induced current: knowledge; CIC2, Conditions to generate induced current: understanding; LL1, Lenz’s Law: knowledge; LL2, Lenz’s Law: understanding; LL3, Lenz’s Law: application; RHR1, Right-hand rule: knowledge; RHR2, Right-hand rule: understanding; FL1, Faraday’s law: knowledge; FL2, Faraday’s law: understanding; FL3, Faraday’s law: application; and FL4, Faraday’s law: integrated application.

As shown in Table 6, if the attribute mastery pattern is all 1 (i.e., 1111), it means that the student reached the highest learning level and does not need to participate in remedial teaching. The proportion of the other 12 classifications was 0.0702–0.3526, indicating that the classification scheme was able to basically stratify students according to the learning level of each knowledge content. However, the distinction between FL3 and FL4 was not obvious. In the attribute mastery pattern related to Faraday’s law (FL1, FL2, FL3, and FL4), the proportion of 1111 was 0.0702, while the proportion of 1111 was 0.3263. This indicates that if students are able to achieve the “Faraday’s Law: Application” level, most of them would also be able to reach the level of “Faraday’s Law: Integrated Application.” In solving Faraday’s law, most students who were able to solve two physical processes would also be able to solve more than two physical processes and correlate other knowledge problems.

### Effect of Multi-level Remedial Teaching

After receiving multi-level remedial teaching (experimental group) and the explanation of the answers to the test questions (control group), both groups completed Form B as the post-test.

**Figure 10:**
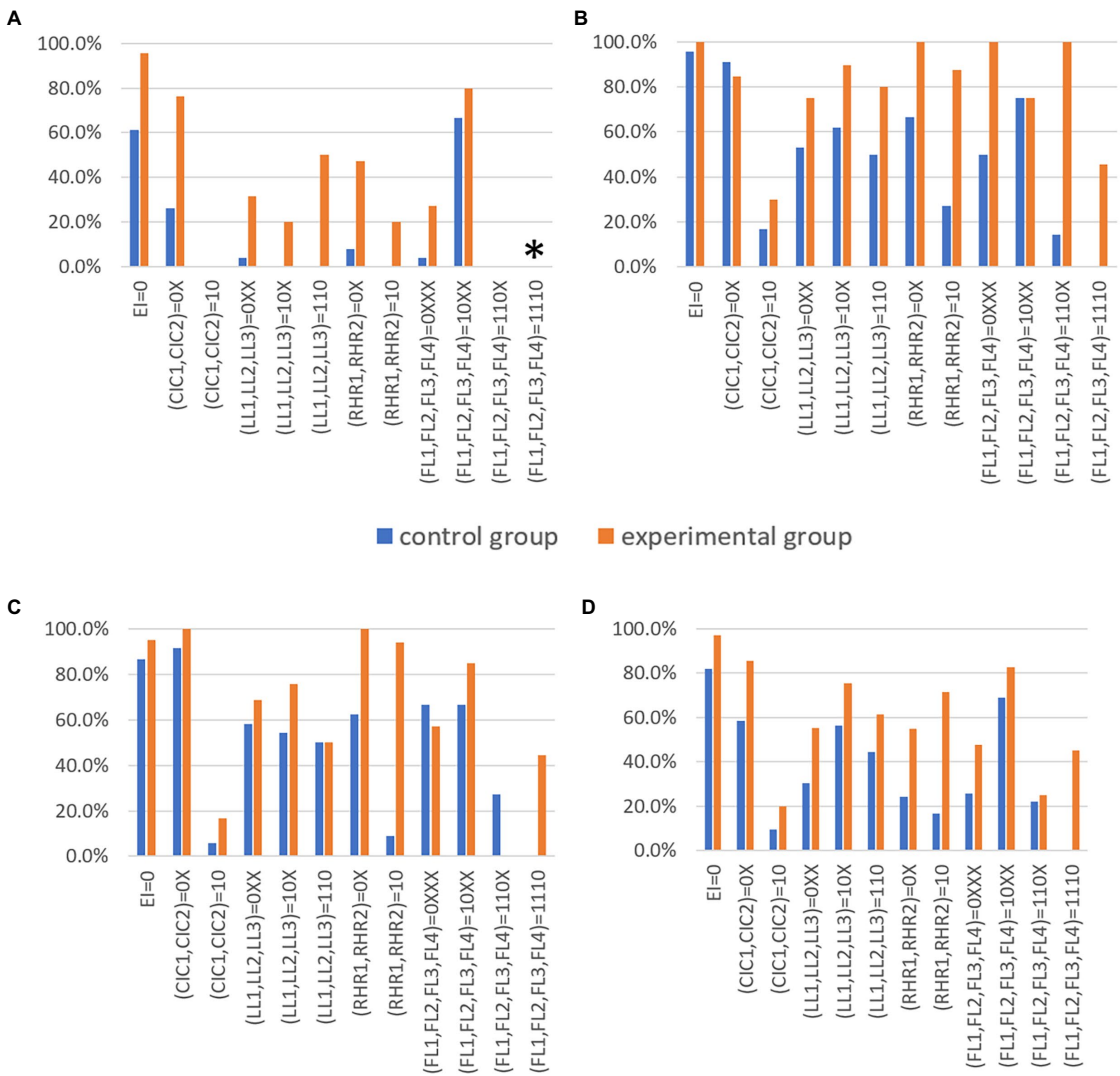
Comparison of the achievement rate of 12 target attribute mastery patterns between the control and experimental groups in schools S1, S2, and S3. **(A)** is a bar graph comparing the achievement rate of 12 target levels between the control and experimental groups of participants at School S1. The achievement rate of (FL1,FL2,FL3,FL4) = (1110) was 0 because no student had mastered the attributive FL3, so no students should regard (FL1,FL2,FL3,FL4) = (1110) as the target attribute mastery. **(B,C)** show seniors at Schools S2 and S3. **(D)** Shows a bar comparison of the average achievement rate of 12 target levels between the control and experimental groups in Schools S1, S2, and S3.

Figures 10A–D show the target attribute mastery pattern achievement rate of 12 attribute mastery pattern classifications in the experimental group and control group in S1, S2, and S3, and the average of the three schools, respectively. Figure 10D shows that both multi-level remedial teaching and the traditional method of explaining answers are able to improve the learning level of students in 12 classifications, but multi-level remedial teaching was found to be more effective and more efficient. The target achievement rates of the experimental group and the control group of the three schools also had their own characteristics. It should be mentioned that we focused on the classification where the experimental group had a greater than 20% target achievement rate compared to the control group. The effect of multi-level remedial teaching on the level of “knowledge” in school S1 was more obvious, but in school S2 and school S3, the difference between the experimental group and the control group of “electromagnetic induction phenomenon: knowledge” and “conditions for generating induced current: knowledge” was not as obvious. The experimental group in the (LL1, LL2, LL3) = (10X) and (110), (RHR1, RHR2) = (0X) and (10), (FL1, FL2, FL3, FL4) = (0XXX), (110X) and (1110) categories of the school S2 had a more obvious effect than the control group. In addition, the experimental group of (RHR1, RHR2) = (0X) and (10), (FL1, FL2, FL3, FL4) = (1110) of school S3 had more obvious effects of remedial teaching. Therefore, these results may be related to the varied levels of the schools.

Since the three schools were of different levels and had large differences between them, the ANOVA of multi-level remedial teaching was not analyzed (Figure 10).

To compare the differences in remedial teaching among the three schools, ANOVA was performed with the method of remedial teaching as the independent variable. The students’ target attribute mastery pattern achievement rate was considered as the dependent variable. The results are shown in Table 7.

**Table 7:** ANOVA results of S1, S2, and S3.

Source of difference	School	SS	df	MS	*F*	value of *p*	F crit
Remedial measures	S1	0.388	1	0.388	4.77	0.0424^*^	4.41
	S2	0.181	1	0.181	1.77	0.197	4.30
	S3	0.557	1	0.557	7.80	0.011^*^	4.301

*SS*, *Sum of squares, df*, *degree of freedom, MS, Mean square, F, Test statistic, and F crit, Critical value of the test*.

* *p* < 0.05.

For the seniors at schools S1 (*F* = 4.77 > F crit = 4.41, *p* = 0.0424 < 0.05) and S3 (*F* = 7.80 > F crit = 4.301, *p* = 0.011 < 0.05), Table 7 reports that different remedial teaching method led to significant differences in the target attribute mastery pattern achievement rate. However, for school S2, the effect of remedial teaching method was not significant (*F* = 1.77 < F crit = 4.30, *p* = 0.197 > 0.05), indicating that there was no significant difference in the target level achievement rate among participants with different remedial methods.

## Discussion and Conclusion

In order to study the effect of multi-level remedial teaching for students, we used the CDA method to develop the EICD test and to carry out the multi-level classification for students on “electromagnetic induction”—a topic that is a part of the usual high school physics curriculum. The EICD test reliability was high, and the consistency of Forms A and B was good; thus, these could be effectively used for the multi-level classification of the students. As for the cognitive errors analyzed from the think-aloud protocols, remedial teaching schemes for the different classified levels were then designed. The results of the experiment showed that the multi-level remedial teaching design used in this study was more effective for the improvement of students’ learning than the traditional way. In a region, the effect of CDA-based multi-level remedial teaching in promoting learning was found to be more significant in high-level and low-level schools, but not in secondary schools. Although this study only used the multi-level remedial teaching design method for one topic in high school physics, the developed multi-level remedial teaching method of assessment, classification, and remedial teaching design can theoretically be used for any high school physics topic. The method designed in this study does not rely on purchasing hardware and software and costs an acceptable amount. For teachers, all progress requires a significant amount of time. However, it enables students to study more efficiently, which benefits teaching efficiency. To further promote teaching efficiency, the completed teaching scheme can be reused or revised for multiple classes.

### Limitations

With the continuous development of research on CDMs, more refined and improved performance models are being developed by researchers, such as a series of longitudinal diagnostic classification models developed by Zhan et al. wherein attribute-level growth may be quantified in a more refined manner (Zhan et al., 2019a, Zhan, 2021; Zhan and He, 2021). A mixed model method can also be considered so that different items in a test are calculated using different models. This is one of the limitations of this study; thus, a model with better fit should be tried in future studies. Second, in order to analyze the cognitive errors of students learning about electromagnetic induction, a limited amount of think-aloud material was collected given the limited time. Hence, it is highly likely that not all cognitive errors were covered. Therefore, teachers on the frontline of teaching may be able to accumulate more comprehensive and accurate cognitive errors during the teaching process, which can then be used to design other multi-level remedial teaching plans. Third, the classification of students in this study was limited to knowledge content and learning level, so future research should focus on improving the cognitive structure and developing potential traits as cognitive attributes.

Although the scheme of multi-level remedial teaching in this study revolved around the topic of electromagnetic induction in high school physics, the method of developing the design scheme may be applied to other topics and other subjects as well. In terms of teaching, the program can be constantly revised and improved to further enhance the teaching effect.

## Data Availability Statement

The original contributions presented in the study are included in the article/Supplemental Material, further inquiries can be directed to the corresponding author.

## Ethics Statement

The studies involving human participants were reviewed and approved by the Committee on Human Research Protection, East China Normal University. Written informed consent to participate in this study was provided by the participants’ legal guardian/next of kin.

## Author Contributions

RH and SP conceived the study. RH developed the cognitive model and the test items. DZ, QH, and RH conducted the think-aloud protocols. DZ, QH, and RH conducted the teaching experiment and collected the test results. RH analyzed the data and wrote the manuscript. ZL and RH revised the manuscript. SP provided technical advice. All authors contributed to the article and approved of the submitted version.

## Funding

This work was supported by the National Education Science Planning Key Projects of the Ministry of Education under Grant DHA170352.

## Conflict of Interest

The authors declare that the research was conducted in the absence of any commercial or financial relationships that could be construed as a potential conflict of interest.

## Publisher’s Note

All claims expressed in this article are solely those of the authors and do not necessarily represent those of their affiliated organizations, or those of the publisher, the editors and the reviewers. Any product that may be evaluated in this article, or claim that may be made by its manufacturer, is not guaranteed or endorsed by the publisher.

## Footnotes

^1^ http://www.psychometrics-studio.cn/
