# Supplementary material for: A Multi-level Remedial Teaching Design Based on Cognitive Diagnostic Assessment: Taking the Electromagnetic Induction as an Example
Source: Front Psychol. 2022 Mar 23;13:851378. doi: 10.3389/fpsyg.2022.851378 (PMC8984267; doi:10.3389/fpsyg.2022.851378)
Supplement: Supplementary file 1 [file Table_1.DOCX]

Supplementary Table S1. The Q-matrix of the Electromagnetic Induction Cognitive Diagnostic Test.

| **Attribute** | **EIP** | **CIC1** | **CIC2** | **LL1** | **LL2** | **LL3** | **RHR1** | **RHR2** | **FL1** | **FL2** | **FL3** | **FL4** |
| --- | --- | --- | --- | --- | --- | --- | --- | --- | --- | --- | --- | --- |
| Item 1 | 1 | 0 | 0 | 0 | 0 | 0 | 0 | 0 | 0 | 0 | 0 | 0 |
| Item 2 | 1 | 0 | 0 | 0 | 0 | 0 | 0 | 0 | 0 | 0 | 0 | 0 |
| Item 3 | 1 | 0 | 0 | 0 | 0 | 0 | 0 | 0 | 0 | 0 | 0 | 0 |
| Item 4 | 0 | 1 | 0 | 0 | 0 | 0 | 0 | 0 | 0 | 0 | 0 | 0 |
| Item 5 | 0 | 1 | 0 | 0 | 0 | 0 | 0 | 0 | 0 | 0 | 0 | 0 |
| Item 6 | 0 | 1 | 0 | 0 | 0 | 0 | 0 | 0 | 0 | 0 | 0 | 0 |
| Item 7 | 0 | 0 | 1 | 0 | 0 | 0 | 0 | 0 | 0 | 0 | 0 | 0 |
| Item 8 | 0 | 0 | 1 | 0 | 0 | 0 | 0 | 0 | 0 | 0 | 0 | 0 |
| Item 9 | 0 | 0 | 1 | 0 | 0 | 0 | 0 | 0 | 0 | 0 | 0 | 0 |
| Item 10 | 0 | 0 | 0 | 0 | 0 | 0 | 1 | 0 | 0 | 0 | 0 | 0 |
| Item 11 | 0 | 0 | 0 | 0 | 0 | 0 | 1 | 0 | 0 | 0 | 0 | 0 |
| Item 12 | 0 | 0 | 0 | 0 | 0 | 0 | 1 | 0 | 0 | 0 | 0 | 0 |
| Item 13 | 0 | 0 | 0 | 0 | 0 | 0 | 0 | 1 | 0 | 0 | 0 | 0 |
| Item 14 | 0 | 0 | 0 | 0 | 0 | 0 | 0 | 1 | 0 | 0 | 0 | 0 |
| Item 15 | 0 | 0 | 0 | 0 | 0 | 0 | 0 | 1 | 0 | 0 | 0 | 0 |
| Item 16 | 0 | 0 | 0 | 1 | 0 | 0 | 0 | 0 | 0 | 0 | 0 | 0 |
| Item 17 | 0 | 0 | 0 | 1 | 0 | 0 | 0 | 0 | 0 | 0 | 0 | 0 |
| Item 18 | 0 | 0 | 0 | 1 | 0 | 0 | 0 | 0 | 0 | 0 | 0 | 0 |
| Item 19 | 0 | 0 | 0 | 0 | 1 | 0 | 0 | 0 | 0 | 0 | 0 | 0 |
| Item 20 | 0 | 0 | 0 | 0 | 1 | 0 | 0 | 0 | 0 | 0 | 0 | 0 |
| Item 21 | 0 | 0 | 0 | 0 | 1 | 0 | 0 | 0 | 0 | 0 | 0 | 0 |
| Item 22 | 0 | 0 | 0 | 0 | 0 | 1 | 0 | 0 | 0 | 0 | 0 | 0 |
| Item 23 | 0 | 0 | 0 | 0 | 0 | 1 | 0 | 0 | 0 | 0 | 0 | 0 |
| Item 24 | 0 | 0 | 0 | 0 | 0 | 1 | 0 | 0 | 0 | 0 | 0 | 0 |
| Item 25 | 0 | 0 | 0 | 0 | 0 | 0 | 0 | 0 | 1 | 0 | 0 | 0 |
| Item 26 | 0 | 0 | 0 | 0 | 0 | 0 | 0 | 0 | 1 | 0 | 0 | 0 |
| Item 27 | 0 | 0 | 0 | 0 | 0 | 0 | 0 | 0 | 1 | 0 | 0 | 0 |
| Item 28 | 0 | 0 | 0 | 0 | 0 | 0 | 0 | 0 | 0 | 1 | 0 | 0 |
| Item 29 | 0 | 0 | 0 | 0 | 0 | 0 | 0 | 0 | 0 | 1 | 0 | 0 |
| Item 30 | 0 | 0 | 0 | 0 | 0 | 0 | 0 | 0 | 0 | 1 | 0 | 0 |
| Item 31 | 0 | 0 | 0 | 0 | 0 | 0 | 0 | 0 | 0 | 0 | 1 | 0 |
| Item 32 | 0 | 0 | 0 | 0 | 0 | 0 | 0 | 0 | 0 | 0 | 1 | 0 |
| Item 33 | 0 | 0 | 0 | 0 | 0 | 0 | 0 | 0 | 0 | 0 | 1 | 0 |
| Item 34 | 0 | 0 | 0 | 0 | 0 | 0 | 0 | 0 | 0 | 0 | 0 | 1 |
| Item 35 | 0 | 0 | 0 | 0 | 0 | 0 | 0 | 0 | 0 | 0 | 0 | 1 |
| Item 36 | 0 | 0 | 0 | 0 | 0 | 0 | 0 | 0 | 0 | 0 | 0 | 1 |

The measurement patterns are represented by a matrix, and the elements in the matrix equal to 0 or 1, indicating that the corresponding attribute is unmeasured or measured in the item, respectively.

EIP=electromagnetic induction phenomenon: knowledge; CIC1=conditions to generate induced current: knowledge; CIC2=conditions to generate induced current: understanding; LL1=Lenz’s law: knowledge; LL2=Lenz’s law: understanding; LL3=Lenz’s law: application; RHR1=right-hand rule: knowledge; RHR2=right-hand rule: understanding; FL1=Faraday’s law of induction: knowledge; FL2=Faraday’s law of induction: understanding; FL3=Faraday’s law of: application; FL4=Faraday’s law: integrated application.

Supplementary Table S2. Interview records about the differences in diagnostic results between the DINA model and the GDM model

| **Participant** | **Attributes** | **DINA result** | **GDM**  **result** | **Interview record** | **More suitable model** |
| --- | --- | --- | --- | --- | --- |
| 1 | EIP | 0 | 1 | Q：Because you got the third question right, you should know that electromagnetic induction produces "electricity". But why did you choose option B for the second question?  A：I think option B is the phenomenon of electromagnetic induction, because a magnetic field is induced around the current. | DINA |
| 1 | CIC1 | 0 | 1 | Q：What was your train of thought when you solved questions 5 and 6?  A：I remember that whenever there is a closed circuit and a cut magnetic field line, there is an induced current. | DINA |
| 3 | CIC1 | 0 | 1 | Q：Please retell your thoughts on answering Questions 5 and 6.  A：I remember a knowledge that cutting the magnetic field lines produces induced currents.  Q：So which one do you think is more confident to answer Question 5 and Question 6?  A：Question 5.  Q：You answered question 5 wrong. A sufficient and necessary condition for the induced current to be generated is the change of the magnetic flux through the closed loop. Conductor cutting magnetic field lines does not necessarily change the magnetic flux. Is it possible that the conductor cuts the magnetic field lines, but the magnetic flux does not change?  A：I got it, the equivalent magnetic field. | DINA |
| 4 | CIC2 | 0 | 1 | Q：Please retell your thoughts on answering Questions 7, 8, and 9.  A：I don't know, it's purely based on feeling.  Q：You got question 8 right, what did you think at the time?  A：I guessed right. | DINA |
| 5 | LL1 | 0 | 1 | Q：What was your thinking when answering Questions 16, 17, and 18?  A：I don't quite understand these three questions, and I don't know how to answer them. | DINA |
| 6 | LL1 | 1 | 0 | Q：Is option B of question 17 correct?  A：Probably wrong. Because Lenz's law says that the magnetic field of the induced current hinders the original magnetic field, the two magnetic fields are in opposite directions. | DINA |
| 7 | RHR1 | 0 | 1 | Q：Question 10 you wrote irregularly, it should be "vertical". You know you should use the Right-hand Rule, right?  A：Yes.  Q：Then why don't you answer the Questions 13, 14, and 15?  A：I know the Right hand Rule is required to answer these three questions, but I don't know how to use it. | GDM |
| 8 | RHR1 | 1 | 0 | Q: What are your thoughts on answering questions 11 and 12?  A：When answering question 11, I remembered that the teacher said that the Right-hand Rule is to determine the direction of the closed induced current.  Q：Why did you choose the Right-hand Ampere Rule for question 12?  A：I made a mistake. The correct answer to Question 12 should be B. | DINA |
| 9 | RHR2 | 0 | 1 | Q：You got question 10 right. What did you think about Questions 11 and 12 at the time?  A：I ruled out options B and D when answering Question 11.  Q：What is the distribution of the magnetic field around the energized straight wire in option B?  A：Use the right hand  Q：Do you know the difference between the Right Hand Rule and the Right-hand Screw Rule?  A：(Silence) | DINA |
| 19 | FL1 | 1 | 0 | Q：You answered questions 26 and 27 correctly. Why is Question 25 answered incorrectly?  A：The formulas for Questions 26 and 27 were memorized by me.  Q：What is the specific meaning of the formula?  A：I have absolutely no idea. | GDM |

Table S2 recorded the interviews of participants with discordant diagnostic results between the DINA model and the GDM model on the formal test. Interviews were organized by the authors and physics teachers at participating schools. In the “Interview Record” column, “Q” represents the interviewers’ question, and “A” represents the student's answer. According to the interview results, it was determined that the DINA model is more suitable for this study.
